# Facilitative plasma membrane transporters function during ER transit

**DOI:** 10.1096/fj.09-146472

**Published:** 2010-08

**Authors:** Hitomi Takanaga, Wolf B. Frommer

**Affiliations:** Carnegie Institution for Science, Department of Plant Biology, Stanford, California, USA

**Keywords:** endoplasmic reticulum, uniporter, sodium-cotransporter, fluoresecence resonance energy transfer, sensor

## Abstract

Although biochemical studies suggested a high permeability of the endoplasmic reticulum (ER) membrane for small molecules, proteomics identified few specialized ER transporters. To test functionality of transporters during ER passage, we tested whether glucose transporters (GLUTs, SGLTs) destined for the plasma membrane are active during ER transit. HepG2 cells were characterized by low-affinity ER transport activity, suggesting that ER uptake is protein mediated. The much-reduced capacity of HEK293T cells to take up glucose across the plasma membrane correlated with low ER transport. Ectopic expression of GLUT1, -2, -4, or -9 induced GLUT isoform-specific ER transport activity in HEK293T cells. In contrast, the Na^+^-glucose cotransporter SGLT1 mediated efficient plasma membrane glucose transport but no detectable ER uptake, probably because of lack of a sufficient sodium gradient across the ER membrane. In conclusion, we demonstrate that GLUTs are sufficient for mediating ER glucose transport *en route* to the plasma membrane. Because of the low volume of the ER, trace amounts of these uniporters contribute to ER solute import during ER transit, while uniporters and cation-coupled transporters carry out export from the ER, together potentially explaining the low selectivity of ER transport. Expression levels and residence time of transporters in the ER, as well as their coupling mechanisms, could be key determinants of ER permeability.—Takanaga, H., Frommer, W. B. Facilitative plasma membrane transporters function during ER transit.

Intracellular membranes play a central role in metabolism by creating subcompartments for specific enzymatic reactions. The subcellular membranes must be able to selectively import and export ions and metabolites. Although it is comparatively easy to isolate some organelles such as mitochondria for studying their transport properties, it is difficult to measure the selectivity of other intracellular compartments such as the endoplasmic reticulum (ER) or the Golgi cisternae in an intact cell. Therefore, most studies of ER transport were carried out in microsomal fractions or in permeabilized cells.

Several studies have suggested that the composition of the ER lumen differs from that of the cytosol with respect to ions and other small molecules. The ER serves as a calcium store, site of synthesis, modification and translocation of proteins, and drug detoxification, as well as a metabolic compartment, *e.g.*, in sterol synthesis. It has been a long-standing debate whether the ER contains a wide spectrum of carriers, whether the translocon represents a nonspecific leak, or whether the ER lipid membrane is more permeable compared to other cellular membranes (1–3). To date, only a few specific ER transporters have been found (4, 5).

Transport of small solutes across the ER membrane is of special relevance in the context of blood glucose homeostasis. In the liver, glucose is produced from glucose-6-phophate (G6P), which derives from glycogenolysis or gluconeogenic precursors. G6P is imported into the ER by a G6P-transporter, designated T1 and encoded by SLC37A4 (6). SLC37A4 is an example of an essential ER transporter, because a deficiency will lead to von Gierke’s disease. Inside the ER lumen, free glucose is released from G6P by an ER membrane G6P phosphatase. The transporters predicted to be necessary for exporting phosphate and glucose into the cytosol were designated T2 and T3, respectively (7, 8). It has long been debated whether the ER of liver cells actually contains a glucose transporter (T3), whether transport is uni- or bidirectional (9), or whether flux is mediated by a vesicular efflux path (10). Proteomics surveys of the ER identified the known transporters involved in calcium and glucose-6-phosphate transport, but did not reveal a significant number of other polytopic membrane proteins that may be involved in other ER transport processes (4, 5).

At least 3 hypotheses could potentially explain glucose export from the ER: *1*) glucose transport is mediated by so-called nonspecific sites (1, 3), *2*) export is mediated by an elusive transporter responsible specifically for ER glucose export, or *3*) plasma membrane glucose transporters of the GLUT family, during their transit to the plasma membrane, are sufficient to mediate glucose export from the ER. Although the genes for both the G6P transporter and the G6P phosphatase have been cloned successfully, and mutations in either of the 2 genes lead to glycogen storage diseases, yet no mutation in a gene corresponding to T3 has been identified (11, 12). This observation is consistent with either hypothesis 1, *i.e.,* nonspecific transport or may be a consequence of redundancy of ER glucose transporters or lethality of mutations in these transporters as suggested in hypotheses 2 or 3.

Förster resonance energy transfer (FRET) nanosensors provide a unique tool enabling quantitative flux analysis with subcellular resolution. FRET calcium sensors have successfully been used to monitor changes in ER calcium levels in response to gallate treatment (13). Using the same concept, it should thus be possible to measure glucose flux across the ER membrane. Genetically encoded FRET-based nanosensors have been developed for a variety of sugars and amino acids. The nanosensors are composed of the bacterial periplasmic binding proteins as a recognition element coupled allosterically to a set of 2 spectral variants of the green fluorescent protein (GFP) as reporter elements (14–17). Conformational changes induced by ligand binding to the recognition element translate into a change in FRET between the attached eCFP and eYFP moieties, permitting noninvasive measurements of analyte levels in living cells (15). To determine analyte levels inside organelles, these genetically encoded nanosensors can be targeted to the respective subcellular compartments (18). To directly monitor glucose flux across the ER membrane, genetically encoded FRET glucose sensors were targeted to the ER lumen by flanking them with an ER signal sequence and a KDEL retention signal. Such ER signal sequences have been used extensively to target heterologous proteins, including GFP variants, to the ER lumen and to retain them in the ER by anchoring *via* the KDEL retention signal (19). In previous work, the use of FRET glucose sensors targeted to the ER of HepG2 cells identified high glucose flux rates across the ER membrane, suggesting the existence of rapid bidirectional high-capacity transport activities for glucose (9).

To monitor ER glucose flux, we used a set of improved cytosolic or ER-targeted FRET glucose sensors covering the low-micro- to medium millimolar affinity range (17, 20). Results obtained with these high-sensitivity sensors showed that at low levels of glucose ER transport is limiting, asymmetric, and saturable. We thus hypothesize that the observed saturable transport activity is mediated by transport proteins in the ER membrane. A search for cells with low endogenous glucose transport activity identified HEK293T cells as a suitable test system for plasma membrane and ER transport. Analysis of HEK293T cells coexpressing GLUT1, -2, -4, or -9 demonstrates that plasma membrane GLUTs are sufficient for mediating high-capacity ER glucose transport while transiting through the ER. In contrast, SGLT1, a sodium-dependent transporter, was unable to mediate efficient ER uptake of glucose. Thus GLUT uniporters may be sufficient for T3 activity during ER transit in liver and thus constitute the long-sought-for T3 component of Arion’s cycle (7).

## MATERIALS AND METHODS

### Cells, DNA constructs, and reagents

HepG2 cells were purchased from the American Type Culture Collection (ATCC; Manassas, VA, USA). HEK293T cells were obtained from Craig Garner’s laboratory (Stanford University, Stanford, CA, USA). To generate a more robust series of sensors, the eYFP fluorophore was exchanged with Venus (21). For quantitative imaging of glucose levels in the cytosol and ER of mammalian cells, a cytosolic and an ER version of the FLIPglu-Δ13V series sensors were generated in pcDNA3.1(+) and pEF/myc/ER (Invitrogen, Carlsbad, CA, USA). The vector pEF/myc/ER provides ER targeting and retention signal. Affinity mutants were generated by site-directed mutagenesis to produce FLIPglu-30μΔ13V (mglB-D154A), FLIPglu-600μΔ13V (mglB-F16A), FLIPglu-3.2mΔ13V (mglB-F16A and S112A) in pcDNA3.1(+), and pEF/myc/ER. Their detection ranges are shown in Supplemental Fig. 1*A*.

To express GLUT1, -2, -4, and -9 in HEK293T cells, ORFs were amplified by RT-PCR from human liver total RNA (Clontech, Palo Alto, CA, USA) and subcloned into pcDNA3.2/v5-DEST (Invitrogen) using an LR reaction (Invitrogen) from GLUTx-pENTR-TOPO (22). GLUT1 mutants were generated with the GeneTailor site-directed mutagenesis system (Invitrogen) using the primers GLUT1- R126H-forward: ttgagatgctgatcctgggcCATttcatcatcg; and GLUT1- R126H-reverse: gcccaggatcagcatctcaaaggacttgcc. Successful clones were verified by DNA sequencing. SGLT1, a generous gift from Dr. Michihiro Kasahara (Teikyo University, Tokyo, Japan), was cloned into *Xho*I and *Eco*RI sites of pcDNA3.1(−).

For fluorescence bleedthrough correction, FLIPglu-30μΔ13V versions with either a defective eCFP (W67G) or a defective Venus (Y67G) were constructed by site-directed mutagenesis using primers (W67G: CTGAAGCACTGCACGCCgggGGTCAGGGTGGTCAC; Y67G: GCGAAGCACTGCAGGCCgccGCCCAGGGTGGTCAC) (FLIPglu-30μΔ13V†eCFP and FLIPglu-30μΔ13V†Venus). Cytosolic and ER expression plasmids as well as control sensors are available from Addgene (Cambridge, MA, USA; http://www.addgene.org). The antibody for GLUT1 was purchased from Abcam (Cambridge, MA, USA); the antibody for actin was from Chemicon (Temecula, CA, USA).

### Cell culture and transfection

HepG2 cells were grown in low-glucose DMEM (Invitrogen) with 10% fetal calf serum, 50 U/ml penicillin, and 50 μg/ml streptomycin (Invitrogen). HEK293T cells were grown in high-glucose DMEM (Invitrogen) with 10% fetal calf serum and 50 U/ml penicillin and 50 μg/ml streptomycin (Invitrogen). Cells were cultured at 37°C and 5% CO_2_. For FRET analysis, transfected cells were cultured on collagen-coated round cover glasses (2.5 mm diameter) in 6-well plates. Cells were transiently transfected at 50–70% confluence using Lipofectamine 2000 reagent (Invitrogen) in Opti-MEM I reduced serum medium (Life Technologies, Gaithersburg, MD, USA). After transfection, cells were cultured for ∼6 h in Opti-MEM, then changed to DMEM. Cell division rates of cells expressing the sensor were comparable to that of cells transfected with the empty vector, demonstrating that expression of the sensor did not affect cell division and growth rate to a significant extent (Supplemental Fig. 2). On d 1 or 2 after transfection, the medium was replaced with fresh medium, and cells were analyzed at room temperature. Cover glasses with the attached cells were mounted in a perfusion chamber (Vacu-Cell, VC-MPC-TW; C&L Instruments, Hershey, PA, USA).

### Image acquisition and FRET analysis

FRET analysis was performed on an inverted fluorescence microscope (DM IRE2; Leica Microsystems, Wetzlar, Germany) with an on-chip multiplication gain QuantEM camera (Roper Scientific, Trenton, NJ, USA) and an ×40 oil-immersion objective (N.A. 1.25–0.75, IMM HC×PL Apo CS; Leica). Dual emission intensity ratios were simultaneously recorded using a DualView unit (Optical Insights, Tucson, AZ, USA) with a dual eCFP/eYFP-ET filter set [high-transmission modified magnetron sputter-coated filter sets ET470/24m (470 indicates emission wavelength in nanometers; 24 indicates bandwidth) and ET535/30; Chroma, Rockingham, VT, USA] and Slidebook 4.2 software (Intelligent Imaging Innovations, Denver, CO, USA). Excitation (excitation filters eCFP ET430/24x, eYFP ET500/20x; Chroma) was provided by a Lambda DG4 (Sutter Instruments, Novato, CA, USA) using 100% xenon lamp output. Images were acquired within the linear detection range of the camera at intervals of 5 s. Quantitative data were derived by pixel-by-pixel integration of regions defined in the ratiometric images. The fluorescence intensity [in arbitrary units (AU)] for the eCFP and Venus emission channels was monitored, both at eCFP and Venus excitation. The FRET index *F*_*c*_*/D* for sensitized emission was calculated from the peak intensity ratios (Venus/eCYP) using background and fluorescence bleedthrough corrections (23). For bleedthrough correction, control sensors with a defective eCFP (FLIPglu-30μΔ13V†eCFP) or a defective Venus (FLIPglu-30μΔ13V†Venus) were monitored and used for correction: the FRET index *F*_*c*_ = *F*_*f*_ − *D*_*f*_ × (*F*_*d*_*/D*_*d*_) − *A*_*f*_ × (*F*_*a*_*/A*_*a*_), where *F*_*f*_ is the sensitized emission (eCFP_ex_/Venus_em_), *D*_*f*_ is the CFP emission from the FRET sensor (eCFP_ex_/eCFP_em_), *A*_*f*_ is the apparent YFP emission from the FRET sensor (Venus_ex_/Venus_em_), *F*_*d*_*/D*_*d*_ = 77.3 ± 0.1%, and *F*_*a*_*/A*_*a*_ = 5.30 ± 0.07%. Depending on the expression level of the nanosensor, exposure times were varied between 150 and 200 ms, EM intensification was varied between 300 and 600, gain was 3, software binning was set to 1. Perfusions were performed with Hanks’ buffered saline (137 mM NaCl, 5.4 mM KCl, 0.3 mM Na_2_HPO_4_, 0.4 mM KH_2_PO_4_, 4.2 mM NaHCO_3_, 1.3 mM CaCl_2_, 0.5 mM MgCl_2_, and 0.4 mM MgSO_4_, pH 7.4) using a computer-controlled 8-channel gravity-flow system equipped with a perfusion pencil (AutoMate Science, Berkeley, CA, USA) attached to the Vacu-Cell perfusion chamber at a flow rate of 1.0 ml/min. The baseline of the recordings was corrected using a third-order polynomial fit of the FRET index measured in the absence of glucose in Matlab (script programmed by Dr. Oliver Schweissgut, Siegen, Germany; http://www.uni-siegen.de/fb11/simtec/software/fret/). Changes in the FRET index for sensors expressed in the cytosol and ER were normalized to the initial value and were measured in perfusion experiments for each compartment. Each set of data comprised at least 2–20 cells in the field of view. Cytosolic and ER glucose concentration was calculated by [*C*_cyt_] or [*C*_ER_] = *K*_*d*_ × (1 − *R*_*t*_)/(*R*_*t*_ − *R*_min_) at steady-state level. *R*_min_ was 0.75 as measured by 0.015% digitonin-treated cells. The concentrations were fitted by Michaelis-Menten kinetics, *C*_max_ × *C*_*c*_/(*C*_0.5_ + *C*_*c*_). The ER concentration was fitted by fixed *C*_0.5_ as the same with cytosolic *C*_0.5_.

### Confocal microscopy

HepG2 cells were transfected transiently with FLIPglu-600Δ13V targeted to either cytosol or ER (22). Cells were imaged 24–48 h after transfection using a Nipkow spinning disk confocal microscope. Incident argon (488 nm) ion laser (Coherent, Inc., Santa Clara, CA, USA) beams were coupled to a modified Yokogawa spinning disk confocal scan head (Yokogawa Electric, Tokyo, Japan; Solamere Technology, Salt Lake City, UT, USA) *via* an acoustical optical tunable filter (Neos, Melbourne, FL, USA). The confocal head was mounted on an inverted microscope (DM IRE2; Leica) equipped with an ×63 glycerol-immersion objective (N.A. 1.3, HC×PL APO, 21°C; Leica) and a motorized Z stage. Fluorescence images (band-pass filters of 525/50 nm for yellow fluorescent protein Venus) were acquired with a cooled on-chip multiplication gain Cascade 512B digital camera (Roper Scientific). Instrumentation was driven using Metamorph 6.1r5 software (Universal Imaging Corp., Downingtown, PA, USA).

### Protein gel blots

HEK293T cells were transiently transfected with GLUT1 in pcDNA3.2/v5-DEST in 6 wells for 48 h. HepG2 or HEK293T cells were suspended in PBS and washed twice and incubated in lysis buffer (10 mM HEPES, pH 7.2; 1 mM EDTA; and protease inhibitors; Roche) for 10 min on ice, then homogenized in sucrose buffer (10 mM HEPES, pH 7.2; 250 mM sucrose; and protein inhibitors) and centrifuged at 100,000 *g* for 1 h in a TL-100 ultracentrifuge with a TLA-100 rotor (Beckman, Fullerton, CA, USA). Protein analysis was carried out by SDS-PAGE. Separated proteins were transferred to a PVDF (Immobilon-P; Millipore, Bedford, MA, USA). The membrane was blocked with 5% skim milk in PBS, incubated with anti-GLUT1 rabbit antibody or anti-actin mouse monoclonal antibody (Chemicon), washed, and then incubated with horseradish peroxidase-linked anti-rabbit IgG or anti-mouse IgG. After washing, membranes were exposed to ECL reagents (Pierce, Rockford, IL, USA), and X-ray film was used to visualize immunoreactive proteins.

### RT-PCR analysis of RNA from HepG2 cells and HEK293T cells

RNA was extracted from HepG2 or HEK293T cells, and first-strand cDNA was produced (Ambion, Austin, TX, USA). cDNA fragments were amplified (2 min at 94°C, 40 cycles of 30 s at 94°C, 30 s at 55°C to 60°C, and 1 min at 72°C; 10 min at 72°C) using *Taq*DNA polymerase and the primers published previously (9). Primers had been designed for annealing to exon sequences with the lowest possible degree of homology between isoforms to distinguish between other GLUT family members. PCR products obtained for GLUTs corresponded to the predicted lengths.

## RESULTS

### Glucose flux across the ER membrane of HepG2 cells

**Figure 1.**
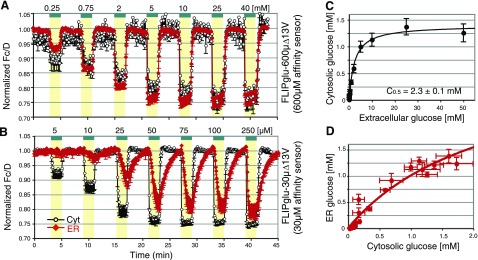
Sugar flux analysis in HepG2 cells with novel set of sensors. *A, B*) FRET analysis of cytosolic and ER glucose levels with FLIPglu-Δ13V series sensors in HepG2 cells. Glucose dynamics were measured in the cytosol (black) and ER (red) of HepG2 cells expressing FLIPglu-600μΔ13V (*A*) and FLIPglu-30μΔ13V (*B*). *C*, *D*) Analysis of the relationship dependence between extracellular concentration and cytosolic concentration (*C*) and the relationship between cytosolic concentration and ER concentration (*D*). Cytosolic and ER glucose concentrations were calculated from [*C*_cyt_] or [*C*_ER_] = *K*_*d*_ × (1 − *R*_*t*_)/(*R*_*t*_ − *R*_*min*_) at steady-state level. The concentrations were fitted by Michaelis-Menten kinetics, *C*_max_ × *C*_*c*_/(*C*_0.5_ + *C*_*c*_). ER concentration was fitted using the same *C*_0.5_ as determined across the plasma membrane. Cells were perfused with different external glucose concentrations. Bars indicate loading time of external glucose concentrations (*A:* 0.25, 0.75, 2, 5, 10, 25, 40 mM; *B:* 5, 10, 25, 50, 75, 100, 250 μM) during continuous perfusion with Hanks’ balanced buffer. Quantitative data were derived from pixel-by-pixel integration of ratiometric images. Fluorescence intensities [arbitrary units (AU)] for individual eCFP (ET470/24m) and Venus (ET535/30m) emission channels were monitored with eCFP excitation (ET430/24x), and the FRET index for Venus and eCYP was determined [*y* axis corresponds to sensitized fluorescence *F*_*c*_*, i.e.,* background and bleedthrough were corrected using Venus excitation (ET500/20x) and normalized to donor emission; note that peak intensities were used and that only the short wavelength emission peak of eCFP was used for donor emission]. FRET images were acquired every 5 s, and cytosolic steady-state glucose levels and accumulation and elimination rates were analyzed. Data are means ± sd (*n*=3–6).

Previous work had identified a high-capacity bidirectional transport activity for glucose across the ER membrane of HepG2 cells (9). At low external glucose supply, a trend of differences between uptake across plasma *vs.* ER membrane were observed; they were, however, statistically not significant (9). Using a FRET sensor with improved dynamic range as well as an improved imaging setup and improved data analysis (17), a reduced glucose steady state in the ER compared to the cytosol was confirmed for the low-concentration range (**Fig. 1*A*** and Supplemental Fig. 1*C*). Correct targeting of FLIPglu-600μΔ13V was verified by confocal microscopy (9). FLIPglu-600μΔ13V was detected in the cytosol, whereas the ER-targeted sensor was detectable in a reticulate structure that colocalized with an ER marker (9) (Supplemental Fig. 1*B*).

The properties of ER transport in the low-concentration range were explored using a high-affinity sensor (FLIPglu-30μΔ13V, *K*_*d*_ 30 μM). When expressed in the cytosol, FLIPglu-30μΔ13V saturated at ∼75 μM external glucose (Fig. 1*B*; black trace) with an apparent *in vivo K*_0.5_ of 10 μM (*K*_0.5_ is defined here as the extracellular concentration at which the sensor is 50% saturated). In the high-affinity range, FLIPglu-30μΔ13V unraveled significant differences between plasma membrane and ER accumulation and elimination kinetics (Fig. 1*B*). Interestingly, in this low-concentration range, the response profile of the ER is asymmetric (influx/efflux; Fig. 1*B*; red trace), most apparent at an external glucose concentration of 250 μM. These data demonstrate that the ER membrane is not freely permeable to glucose but contains low affinity uptake and efflux systems. The steady-state profile was saturable with a *C*_0.5_ of 2.3 ± 0.1 mM (*C*_0.5_ is defined here as the extracellular concentration at which the cytosolic glucose concentration is 50% saturated; corresponding to ∼0.65 mM in the cytosol; cytosolic levels were estimated using the assumption that the *in vivo K*_*d*_ of the sensor is equal to the *in vitro K*_*d*_) (Fig. 1*C* and **Table 1**). ER glucose accumulation was also saturable and could be fitted with the same parameters as for the cytosolic accumulation, an observation that may suggest similar properties for plasma and ER membrane transport (Fig. 1*D*).

### Low endogenous glucose uptake activity in HEK293T cells

**Figure 2.**
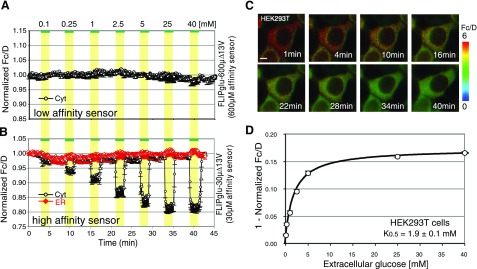
FRET analysis of glucose flux with FLIPglu-Δ13V series sensors in the cytosol or ER of HEK293T cells. *A*, *B*) Glucose dynamics were measured in HEK293T cells coexpressing FLIPglu-600μΔ13V (*A*) or FLIPglu-30μΔ13V (*B*) either in the cytosol (black trace) or ER (red trace). Note that cytosol and ER traces are ratios. *C*) Images of an individual cell using pseudocolors for the cytosolically expressed sensor FLIPglu-30μΔ13V (data shown in *B*). Scale at right corresponds to ratio (normalized *F*_*c*_*/D*). *D*) Relationship dependence between extracellular concentration and normalized *F*_*c*_*/D.* FRET images were acquired, and data were analyzed as in Fig. 1. Data are means ± sd (*n*=10–20).

To investigate whether the ability to move glucose across the ER membrane is specific to the liver cell line, glucose flux was analyzed in cell lines from other origins. The response of glucose FRET sensors, namely, the *in vivo K*_0.5_, varied slightly, but was generally similar for COS-7 (24), HepG2 (9, 22), NIH3T3, CHO, and PC12 cells when using the high-sensitivity sensor FLII^12^Pglu-700μδ6 (22) (data not shown). In contrast, HEK293T cells showed no significant response to external glucose (0.1–40 mM, data not shown). To characterize the apparent lack of glucose accumulation in HEK293T cells in more detail, cells were transfected with FLIPglu-600μΔ13V (**Fig. 2*A***) and FLIPglu-30μΔ13V (Fig. 2*B*, *C*). Cells expressing FLIPglu-600μΔ13V in the cytosol did not show a significant response even at high external supply (40 mM; Fig. 2*A*). In contrast, the high-affinity FLIPglu-30μΔ13V detected a concentration-dependent accumulation of glucose in the cytosol (Fig. 2*B*; black trace). Saturation of FLIPglu-30μΔ13V occurred only at external glucose levels exceeding ∼25 mM in HEK293T cells. Apparently, cytosolic glucose levels are extremely low in HEK293T cells (Fig. 2*D*; cytosolic concentration 30 μM at *K*_0.5_ = 1.9±0.1 mM). This steep glucose gradient across the plasma membrane must either be caused by low uptake capacity or high metabolism of glucose relative to the other cell lines. Consistent with the low glucose uptake rates, RT-PCR indicates that GLUTs 1–7, 9–11, 13, and 14 are expressed at lower levels compared to HepG2 cells (Supplemental Fig. 3).

If ER glucose transport were caused by unspecific leakage (1, 3), the rate of ER uptake and release should be comparable in HEK293T and HepG2 cells at corresponding cytosolic levels. In HepG2 cells, significant ER uptake was observed with FLIPglu-30μΔ13V when cytosolic levels reached ∼50 μM (Fig. 1*B*; ∼75% saturation at the external supply 25 μM glucose, red trace), whereas glucose accumulation was undetectable in HEK293T cells expressing FLIPglu-30μΔ13V (Fig. 2*B*; red trace) at comparable intracellular glucose levels. Thus the low plasma membrane transport activity in HEK293T cells correlates with a low capacity for ER glucose uptake (relative to HepG2 cells), suggesting genetic differences in ER permeability between the 2 cell types and supporting the notion that transport is protein mediated. Moreover, the correlation between ER and plasma membrane transport activity may suggest that the plasma membrane GLUTs may be responsible for the ER transport activity.

### Induction of glucose transport in HEK293T cells expressing GLUTs

**Figure 3.**
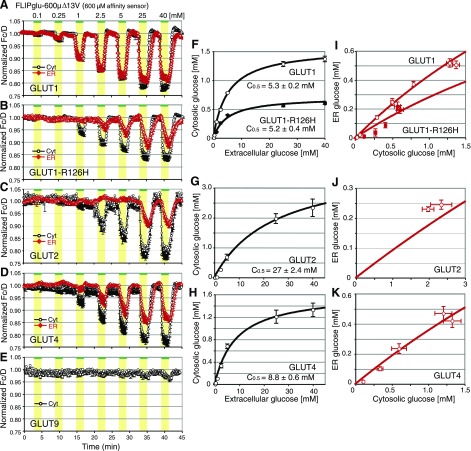
FRET analysis of glucose flux using FLIPglu-600μΔ13V in the cytosol or ER of HEK293T cells expressing GLUT transporters. Glucose dynamics were measured in the cytosol (black trace) or ER (red trace). *A–E*) Glucose dynamics in HEK293T cells coexpressing either GLUT1 (*A*), GLUT1-R126H (*B*), GLUT2 (*C*), GLUT4 (*D*), or GLUT9 (*E*) with FLIPglu-600μΔ13V. *F–H*) Analysis of relationship dependence between extracellular concentration and cytosolic concentration: GLUT1 and GLUT1-R126 (*F, H*); GLUT2 (*G*); GLUT4 (*H*). *I–K*) Analysis of relationship between cytosolic concentration and ER concentration: GLUT1 and GLUT1-R126H (*I*); GLUT2 (*J*); GLUT4 (*K*). Cells were perfused with different external glucose concentrations. FRET images were acquired, and data were analyzed as in Fig. 1. Data are means ± sd (*n*=8–36).

To manipulate plasma membrane transport rates in HEK293T cells, the FRET glucose sensor FLIPglu-600μΔ13V was coexpressed with the glucose facilitators GLUT1 and the mutant GLUT1-R126H, as well as GLUT2, -4, and -9. In contrast to HEK293T cells expressing FLIPglu-600μΔ13V only (Fig. 2*A*), cells coexpressing GLUTs efficiently accumulated glucose in the cytosol (**Fig. 3***A*–*E*; black trace). In GLUT1-expressing cells, cytosolic glucose levels reached the *K*_*d*_ of the sensor of ∼600 μM at an external supply of ∼1 mM glucose (Fig. 3*A*), whereas in the absence of GLUT1, the same external glucose concentration led to a steady-state level of only 30 μM (Fig. 2*B*). Coexpression of the high-affinity sensor FLIPglu-30μΔ13V with GLUT1 led to almost full saturation at the lowest external supply tested of 100 μM glucose (Supplemental Fig. 4*B*). Metabolic conversion rates were low in HEK293T cells as evidenced by only a slow rate of glucose elimination from the cytosol in the presence of the GLUT inhibitor cytochalasin B (Supplemental Fig. 5); thus glucose accumulation in HEK293T cells is dominated by GLUT activity. To exclude the possibility that GLUT1 expression induces an endogenous ER transporter, a GLUT1 mutant (GLUT1-R126H) with reduced *C*_max_ (without change in *C*_0.5_, Fig. 3*B*, black trace; *F*), was expressed in HEK293T cells (25, 26). GLUT1-R126H expression led to reduced transport activity at the plasma membrane, demonstrating that accumulation rates depend directly on GLUT1 activity.

Coexpression of GLUT2 and GLUT4 with FLIPglu-600μΔ13V leads to very different kinetics of glucose accumulation and elimination as compared to GLUT1 (Fig. 3*C*, *D**;* black trace), consistent with differences in affinity and *C*_max_ (GLUT1, *K*_*m*_ ≈1–3 mM; GLUT2, *K*_*m*_ ≈17 mM; GLUT4, *K*_*m*_ ≈5 mM; ref. 27; Fig. 3*F*–*H* and Table 1). Consistent with the low affinity of GLUT2, a significant delay of the elimination phase was observed at high external glucose supply (Fig. 3*C* and Supplemental Fig. 4*C*). GLUT9, a low-capacity glucose transporter (22), did not increase the steady-state levels when FLIPglu-600μΔ13V was used (Fig. 3*E*), but increased steady-state levels in the high affinity range (Supplemental Fig. 4*E*), further supporting the notion that GLUT9 can transport glucose (22). GLUT9-GFP showed plasma membrane localization comparable to that of GLUT1-GFP (Supplemental Movies 1 and 2).

### Induction of ER glucose transport in HEK293T cells expressing GLUTs

To test whether the residence time of GLUTs in the ER is sufficient to affect the permeability of the ER membrane for glucose, HEK293T cells coexpressing GLUT1, the mutant GLUT1-R126H, GLUT2, or GLUT4 with an ER-targeted FLIPglu-600μΔ13V were analyzed (Fig. 3*A*–*D**;* red trace). Coexpression of FLIPglu-600μΔ13V with GLUT1, -2, or -4 induced high-capacity ER glucose uptake. The mutant GLUT1-R126H showed a reduced *C*_max_ for ER accumulation (Fig. 3*B*, red trace; *I;* and Table 1). As observed above for the plasma membrane uptake, the kinetics of glucose flux across the ER membrane in HEK293T cells expressing GLUT2 or GLUT4 (Fig. 3*C*, *D*, red trace; *J;* *K*) were very different from those in cells expressing GLUT1 (Fig. 3*A*, red trace; *I*). In both cases, ER uptake was limiting and accumulation, and elimination rates were asymmetric (Fig. 3*C*, *D**;* red trace).

**Figure 4.**
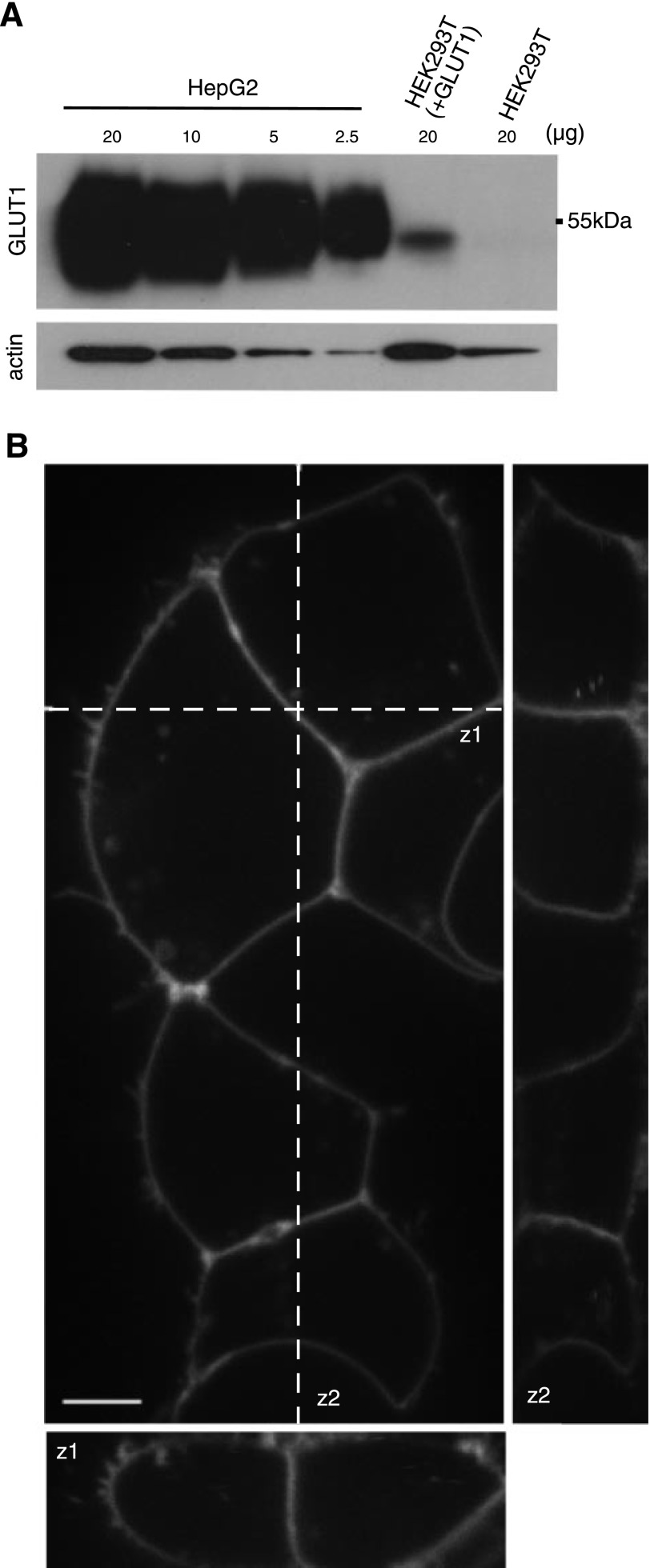
Protein level of GLUT1 between HepG2 cells and HEK293T cells expressing GLUT1 and the localization of GLUT1-GFP fusion protein. *A*) Protein gel blot analysis of GLUT1 levels in HepG2 cells as compared to HEK293T cells expressing GLUT1. Microsomal membrane fractions were extracted and separated by SDS-PAGE (4–20% gradient gel). Actin levels were measured as a control. *B*) Localization of GLUT1-GFP in HEK293T cells. GLUT1-GFP fusion protein was expressed in HEK293T cells, and GFP-derived fluorescence was analyzed by confocal microscopy. Z sections (71 images, 10.3 μm) are marked as sections z_1_ and z_2_. Scale bar = 5 μm (41.5 pixels).

Overexpression of GLUTs could lead to mistargeting and thereby affect glucose accumulation in the ER. However, GLUT1 levels in HEK293T cells expressing GLUT1, as determined by protein gel blots, were at least an order of magnitude lower than those found in HepG2 cells (**Fig. 4*A***). Careful confocal analysis of a GLUT1-GFP fusion protein (C-terminal fusion) expressed from the same promoter as used for GLUT expression above in HEK293T cells did not show detectable accumulation of GLUT1 and GLUT9 in the ER (Fig. 4*B* and Supplemental Movies 1, 2). Comparable results were obtained with an N-terminal GFP fusion to GLUT1 (data not shown). These data suggest that the GLUT1-mediated glucose transport across the ER membrane does not represent an artifact due to overaccumulation in the cell. Thus probably because of the low ER volume; the residence time of GLUTs on their path to the plasma membrane suffices for high-capacity glucose transport.

### SGLT1 does not confer ER glucose transport activity in HEK293T cells

**Figure 5.**
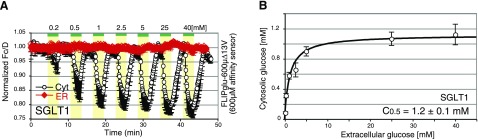
*A*) FRET analysis of glucose flux using FLIPglu-600μΔ13V in the cytosol or ER of HEK293T cells expressing SGLT1. *B*) Relationship dependence between extracellular concentration and cytosolic concentration, analyzed as in Fig. 1*B*. Glucose dynamics were measured in the cytosol (black trace) or ER (red trace) in HEK293T cells coexpressing SGLT1 with FLIPglu-600μΔ13V. FRET images were acquired, and data were analyzed as in Fig. 1. Data are means ± sd (*n*=25–30).

The hypothesis that transporters can contribute to ER membrane conductivity predicts that although facilitative transporters can mediate ER transport, coupled transporters may not be functional if the appropriate gradients are not established. Since the ER is thought to lack a Na^+^ gradient (28), and since the ER membrane potential is predicted to work against cation accumulation in the ER lumen (29), Na^+^ cotransporters may not be capable of efficient ER uptake but only able to efflux glucose from the ER. HEK293T cells expressing the Na^+^-dependent glucose transporter SGLT1 mediated efficient uptake of glucose across the plasma membrane when perfused with Hanks’ buffer containing ∼140 mM Na^+^ (**Fig. 5*A***; black trace). In contrast, HEK293T cells coexpressing SGLT1 along with the ER-localized glucose nanosensor FLIPglu-600μΔ13V showed no significant glucose responses in the ER (Fig. 5*A*; red trace). The proposed capacity of SGLT to efflux glucose from the ER is plausible but was not tested.

Together, these results provide evidence that GLUT family members, in contrast to the Na^+^-dependent SGLT1, are sufficient for mediating glucose influx across the ER membrane during their transit through the ER compartment, while both GLUTs and SGLTs could be responsible for glucose efflux from the ER. Our findings suggest that the properties of the transporters (*i.e.*, ion coupling) and their retention time in the ER should be important determinants of the capacity and kinetics of transport across the ER membrane.

## DISCUSSION

### Activity of transporters during ER transit

Among the cellular compartments, ER and Golgi are unique in that they serve multiple functions, namely, as metabolic compartments and as the secretory pathway for protein translocation. Therefore, ER and Golgi contain resident enzymes and transporters, as well as enzymes and transporters *en route* to their final destination, *i.e.*, lysosomes, vacuole, plasma membrane, and the extracellular space. When these proteins are functional during transit and appropriate substrates are available, they will contribute to metabolism and signaling along the translocation path. The contribution to metabolism will depend on their transit times and the availability of cofactors. This apparently may complicate control over reactions occurring in these compartments. If this hypothesis was correct, ER and Golgi would need “specific” enzymes and transporters only for activities that have insufficient capacity or are unavailable. Transporters use variety of mechanisms: primary active, secondary active, and facilitative. Primary active transporters require ATP, secondary active transporters use ion gradients and membrane potential, whereas uniporters facilitate transport along a substrate gradient. When a membrane potential exists, transport of charged compounds will also be affected by the electrochemical potential. Based on the presence of the ATPase on the cytosolic side of the membrane, primary active transporters such as ATPases are expected to be able to import solutes into the lumen of the ER (30). Since uniport of uncharged molecules is independent of ion gradients and membrane potential, uniporters are also predicted to be fully functional during ER transit. The GLUT uniporters have been shown to be functional when trapped in the ER of yeast (31). In contrast, because of the absence of a significant sodium gradient (32), sodium-coupled transporters such as SGLTs are not expected to be functional in import into the ER. Although experimental evidence for the ER membrane potential is lacking, theoretical considerations suggest that the ER lumen contains excess positive charges and thus a negative membrane potential (29). A negative membrane potential would also inhibit SGLT1 import activity but would stimulate efflux activity. Moreover, the affinity of SGLT1, which functions in cellular import of glucose, on the cytosolic side for glucose is very low, further limiting the capacity of this protein to import glucose into the ER (33). We thus hypothesize that a subset of transporters, specifically uniporters for uncharged molecules such as glucose, as well as coupled transporters and ATPases are functional during transit through the ER and thus may contribute to metabolic processes in the ER. Due to the high surface-to-volume ratio, relatively few transporters would be sufficient for high-capacity import of solutes.

The low number of ER-specific transporters found in proteomics studies is consistent with this hypothesis (4). Potentially, our transit activity hypothesis could be expanded to Golgi transport, *e.g.*, to supply glucose for lactose biosynthesis during lactation.

### Efflux of glucose from the ER lumen in liver cells

Mobilization of glycogen stored in the liver is crucial for maintenance of blood glucose levels. The final steps of glucose production from glycogen involve import of G6P into the ER and its dephosphorylation to glucose by the activity of a luminal, membrane-bound phosphatase. Both steps are essential, because genetic defects in either step lead to glycogen-storage disease type I (von Gierke’s disease) (12). However, the mechanism responsible for the release of glucose from the ER, tentatively named T3, is still unknown. Here, using an optimized set of FRET glucose sensors targeted to the ER lumen, we provide evidence that facilitative GLUTs are sufficient to sustain high-capacity transport of glucose across the ER membrane. Although not experimentally verified, we also hypothesize that SGLTs can contribute to glucose efflux, but not to glucose influx into the ER. We found that ER transport capacity correlated with plasma membrane transport activity when various cell lines were compared. We also observed at high glucose concentrations, the ER membrane of HEPG2 cells showed high-capacity glucose influx and efflux. At low levels, the new use of the new high-affinity FRET sensor FLIPglu-30μΔ13V allowed detection of restrictions in uptake and efflux capacity, suggesting the presence of a protein-mediated glucose transport activity. We also developed HEK293T cells as a system suitable for examining the role of plasma membrane glucose transporters because of the low intrinsic rate of cellular glucose uptake relative to catabolic activity in these cells. Moreover, expression of GLUT1, -2, or -4 led to a significant increase in glucose flux across both the plasma membrane and ER membrane. The characteristics of glucose accumulation and elimination in both compartments on alterations of extracellular glucose supply differed between GLUT family members. Consistent with this finding, the characteristics found in the ER of HepG2 cells (Fig. 1*A*), which express mostly GLUT1 (22) was similar to that of HEK293T cells expressing GLUT1 (Fig. 3*A*). Our study indicates that plasma membrane transporters transiting through the ER during their biosynthesis and subcellular targeting are sufficient for high-capacity transport.

Because liver cells express several members of a subgroup of GLUT members (22), it is conceivable that multiple GLUTs contribute to the observed T3 activity. FRET analysis of HepG2 cells transfected with siRNAs directed against members of the glucose uniporter family (GLUTs; SLC2 gene family) had shown that GLUT1 and GLUT9 are the major contributors to plasma membrane uptake (22); however, only GLUT1 siRNA disrupted glucose accumulation in the ER (Supplemental Fig. 6). This latter result suggests that T3 is not mediated by a single GLUT isoform that is specifically retained in the ER. The importance of the plasma membrane activity of GLUT for liver function, together with the redundancy, is in line with the lack of glycogen storage disease cases linked to a T3 deficiency. GLUT2-knockout mice accumulate glycogen, as do human patients with Fanconi-Bickel syndrome caused by defects in GLUT2, the major GLUT expressed in liver (34–36). Thus, it is likely that GLUT2 contributes to glucose transport across ER and/or plasma membrane.

The inability of SGLT1 to reconstitute ER glucose import may be due to the lack of a suitable sodium gradient (32) and a sufficient membrane potential required for efficient glucose import, or due to shorter residence times compared to GLUTs, or due to the low-affinity binding site presented on the cytosolic side of the transporter (33). Nevertheless, our data do not exclude the possibility that SGLTs can also contribute to the physiologically relevant glucose efflux in liver cells.

The data presented, however, do not unequivocally prove that GLUTs are necessary for ER transport. Direct proof for their role in ER efflux is technically difficult, because inhibition of GLUT1 expression blocks cellular glucose uptake (22). Apparently, the loss of uptake at the plasma membrane prevents analysis of ER accumulation (Supplemental Fig. 6). Glucose provided *via* gluconeogenesis in liver slices of GLUT2-knockout mice or microinjection of glucose into liver cells may provide means to test the hypothesis presented here (37).

Based on our findings for facilitative glucose transporters, we propose that the apparent promiscuity of the ER membrane results from the activity of both ER-resident transporters, such as G6PT and IP_3_ receptor (ER calcium channel), as well as the activity of a wide variety of transporters transiting through the ER on their way to the Golgi, various vesicle populations, or the plasma membrane. As for other membranes, the uptake of xenobiotica by the ER (1) may be explained by ability of these compounds to be transported by these transport proteins (38). The amount of protein produced at the ER membrane, its residence time in the ER, and its transport mechanism (*e.g.*, uniport *vs.* primary or secondary active transport) are thus expected to be important determinants of ER membrane permeability and selectivity.

**Table 1.** Parameters obtained with FRET sensors for plasma membrane and ER concentration differences for the different cell types expressing various glucose transporters

Cell type	*C*_0.5_ (mM)	*K_m_* (mM)	Cytosolic *C*_max_ (mM)	ER *C*_max_ (mM)
HepG2	2.3 ± 0.1	—	1.4 ± 0.02	3.3 ± 0.03
GLUT1/HEK293T	5.3 ± 0.2	∼3 (27)	1.5 ± 0.02	3.2 ± 0.04
GLUT1-R126H/HEK293T	5.2 ± 0.4	∼3 (39)	0.71 ± 0.02	1.7 ± 0.1
GLUT2/HEK293T	27.0 ± 2.4	∼17 (27)	4.0 ± 0.2	2.7 ± 0.1
GLUT4/HEK293T	8.8 ± 0.6	∼5 (27)	1.6 ± 0.03	3.4 ± 0.1
SGLT1/HEK293T	1.2 ± 0.1	∼1 (40)	1.1 ± 0.02	ND

*C*_0.5_ and cytosolic and ER *C*_max_ of HepG2 were calculated from Fig. 1*C*, *D. C*_0.5_ and cytosolic and ER *C*_max_ of HEK293T/GLUTs were calculated from Fig. 3*F–K*. *C*_0.5_ and cytocolic *C*_max_ of HEK293T/SGLT1 were calculated from Fig. 5*B*. *K_m_* values were previously reported. *C*_0.5_, extracellular concentration at which cytosolic glucose concentration is 50% saturated; *K_m_*, affinity of transporters as determined in references cited; *C*_max_, maximal glucose concentration reached in respective compartments; ND, not determined. Data are means ± SE.

## Supplementary Material

Supplemental Data

## References

[1] Le Gall S., Neuhof A., Rapoport T. (2004) The endoplasmic reticulum membrane is permeable to small molecules. Mol. Biol. Cell 15, 447–455 14617815 10.1091/mbc.E03-05-0325 PMC329208

[2] Csala M., Banhegyi G., Benedetti A. (2006) Endoplasmic reticulum: a metabolic compartment. FEBS Lett. 580, 2160–2165 16580671 10.1016/j.febslet.2006.03.050

[3] Lizak B., Csala M., Benedetti A., Banhegyi G. (2008) The translocon and the non-specific transport of small molecules in the endoplasmic reticulum (review). Mol. Membr. Biol. 25, 95–101 18307097 10.1080/09687680701670481

[4] Gilchrist A., Au C. E., Hiding J., Bell A. W., Fernandez-Rodriguez J., Lesimple S., Nagaya H., Roy L., Gosline S. J., Hallett M., Paiement J., Kearney R. E., Nilsson T., Bergeron J. J. (2006) Quantitative proteomics analysis of the secretory pathway. Cell 127, 1265–1281 17174899 10.1016/j.cell.2006.10.036

[5] Gao B. B., Stuart L., Feener E. P. (2008) Label-free quantitative analysis of one-dimensional PAGE LC/MS/MS proteome: application on angiotensin II-stimulated smooth muscle cells secretome. Mol. Cell. Proteomics 7, 2399–2409 18676994 10.1074/mcp.M800104-MCP200 PMC2596345

[6] Méchin M. C., van de Werve G. (2000) Glucose-6-phosphate transporter and receptor functions of the glucose-6-phosphatase system analyzed from a consensus defined by multiple alignments. Proteins Struct. Func. Genet. 41, 164–172 10.1002/1097-0134(20001101)41:2<164::aid-prot20>3.0.co;2-2 10966570

[7] Arion W. J., Lange A. J., Walls H. E., Ballas L. M. (1980) Evidence for the participation of independent translocation for phosphate and glucose-6-phosphate in the microsomal glucose-6-phosphatase system: interactions of the system with orthophosphate, inorganic pyrophosphate, and carbamyl phosphate. J. Biol. Chem. 255, 10396–10406 6253473

[8] Foster J. A., Nordlie R. C. (2002) The biochemistry and molecular biology of the glucose-6-phosphatase system. Exp. Biol. Med. 227, 601–608 10.1177/153537020222700807 12192101

[9] Fehr M., Takanaga H., Ehrhardt D. W., Frommer W. B. (2005) Evidence for high-capacity bidirectional glucose transport across the endoplasmic reticulum membrane by genetically encoded fluorescence resonance energy transfer nanosensors. Mol. Cell. Biol. 25, 11102–11112 16314530 10.1128/MCB.25.24.11102-11112.2005 PMC1316956

[10] Hosokawa M., Thorens B. (2002) Glucose release from GLUT2-null hepatocytes: characterization of a major and a minor pathway. Am. J. Physiol. 282, E794–E801 10.1152/ajpendo.00374.2001 11882499

[11] Chen S. Y., Pan C. J., Nandigama K., Mansfield B. C., Ambudkar S. V., Chou J. Y. (2008) The glucose-6-phosphate transporter is a phosphate-linked antiporter deficient in glycogen storage disease type Ib and Ic. FASEB J. 22, 1–8 18337460 10.1096/fj.07-104851

[12] Matern D., Seydewitz H. H., Bali D., Lang C., Chen, Y. T. (2002) Glycogen storage disease type I: diagnosis and phenotype/genotype correlation. Eur. J. Pediatr. 161(Suppl. 1), S10–S19 12373566 10.1007/s00431-002-0998-5

[13] Palmer A. E., Jin C., Reed J. C., Tsien R. Y. (2004) Bcl-2-mediated alterations in endoplasmic reticulum Ca^2+^ analyzed with an improved genetically encoded fluorescent sensor. Proc. Natl. Acad. Sci. U. S. A. 101, 17404–17409 15585581 10.1073/pnas.0408030101 PMC535104

[14] Fehr M., Frommer W. B., Lalonde S. (2002) Visualization of maltose uptake in living yeast cells by fluorescent nanosensors. Proc. Natl. Acad. Sci. U. S. A. 99, 9846–9851 12097642 10.1073/pnas.142089199 PMC125039

[15] Fehr M., Lalonde S., Lager I., Wolff M. W., Frommer W. B. (2003) In vivo imaging of the dynamics of glucose uptake in the cytosol of COS-7 cells by fluorescent nanosensors. J. Biol. Chem. 278, 19127–19133 12649277 10.1074/jbc.M301333200

[16] Lager I., Fehr M., Frommer W. B., Lalonde, S. (2003) Development of a fluorescent nanosensor for ribose. FEBS Lett. 553, 85–89 14550551 10.1016/s0014-5793(03)00976-1

[17] Deuschle K., Okumoto S., Fehr M., Looger L. L., Kozhukh L., Frommer W. B. (2005) Construction and optimization of a family of genetically encoded metabolite sensors by semirational protein engineering. Protein Sci. 14, 2304–2314 16131659 10.1110/ps.051508105 PMC2253473

[18] Fehr M., Lalonde S., Ehrhardt D. W., Frommer W. B. (2004) Live imaging of glucose homeostasis in nuclei of COS-7 cells. J. Fluoresc. 14, 603–609 15617267 10.1023/b:jofl.0000039347.94943.99

[19] Lippincott-Schwartz J., Presley J., Phair R., Hirschberg K. (2000) Secretory protein trafficking in single live cells. Biophys. J. 78, 138A–138A

[20] Deuschle K., Fehr M., Hilpert M., Lager I., Lalonde S., Looger L. L., Okumoto S., Persson J., Schmidt A., Frommer W. B. (2005) Genetically encoded sensors for metabolites. Cytometry A 64, 3–9 15688353 10.1002/cyto.a.20119 PMC2752217

[21] Nagai T., Ibata K., Park E. S., Kubota M., Mikoshiba K., Miyawaki A. (2002) A variant of yellow fluorescent protein with fast and efficient maturation for cell-biological applications. Nat. Biotechnol. 20, 87–90 11753368 10.1038/nbt0102-87

[22] Takanaga H., Chaudhuri B., Frommer W. B. (2008) GLUT1 and GLUT9 as major contributors to glucose influx in HepG2 cells identified by a high sensitivity intramolecular FRET glucose sensor. Biochim. Biophys. Acta 1778, 1091–1099 18177733 10.1016/j.bbamem.2007.11.015 PMC2315637

[23] Vanderklish P. W., Krushel L. A., Holst B. H., Gally J. A., Crossin K. L., Edelman, G. M. (2000) Marking synaptic activity in dendritic spines with a calpain substrate exhibiting fluorescence resonance energy transfer. Proc. Natl. Acad. Sci. U. S. A. 97, 2253–2258 10688895 10.1073/pnas.040565597 PMC15787

[24] Fehr M., Okumoto S., Deuschle K., Lager I., Looger L. L., Persson J., Kozhukh L., Lalonde S., Frommer W. B. (2005) Development and use of fluorescent nanosensors for metabolite imaging in living cells. Biochem. Soc. Trans. 33, 287–290 15667328 10.1042/BST0330287

[25] Brockmann K., Wang D., Korenke C. G., von Moers A., Ho Y. Y., Pascual J. M., Kuang K., Yang H., Ma L., Kranz-Eble P., Fischbarg J., Hanefeld F., De Vivo D. C. (2001) Autosomal dominant Glut-1 deficiency syndrome and familial epilepsy. Ann. Neurol. 50, 476–485 11603379 10.1002/ana.1222

[26] Pascual J. M., Wang D., Yang R., Shi L., Yang H., De Vivo D. C. (2008) Structural signatures and membrane helix 4 in GLUT1: inferences from human blood-brain glucose transport mutants. J. Biol. Chem. 283, 16732–16742 18387950 10.1074/jbc.M801403200 PMC2423257

[27] Uldry M., Thorens B. (2004) The SLC2 family of facilitated hexose and polyol transporters. Pflügers Arch. 447, 480–489 12750891 10.1007/s00424-003-1085-0

[28] Li Y. X., Keizer J., Stojilkovic S. S., Rinzel J. (1995) Ca2+ excitability of the ER membrane: an explanation for IP3-induced Ca^2+^ oscillations. Am. J. Physiol. 38, C1079–C1092 10.1152/ajpcell.1995.269.5.C1079 7491895

[29] Burdakov D., Petersen O. H., Verkhratsky A. (2005) Intraluminal calcium as a primary regulator of endoplasmic reticulum function. Cell Calcium 38, 303–310 16076486 10.1016/j.ceca.2005.06.010

[30] Guerra G., Petrov V. V., Allen K. E., Miranda M., Pardo J. P., Slayman C. W. (2007) Role of transmembrane segment M8 in the biogenesis and function of yeast plasma-membrane H(+) -ATPase. Biochim. Biophys. Acta 1768, 2383–2392 17573037 10.1016/j.bbamem.2007.04.029 PMC2267258

[31] Kasahara T., Kasahara M. (1996) Expression of the rat GLUT1 glucose transporter in the yeast *Saccharomyces cerevisiae*. Biochem. J. 315(Pt. 1), 177–182 8670104 10.1042/bj3150177 PMC1217168

[32] Hooper G., Dick D. A. (1976) Nonuniform distribution of sodium in the rat hepatocyte. J. Gen. Physiol. 67, 469–474 1271040 10.1085/jgp.67.4.469 PMC2214917

[33] Eskandari S., Wright E. M., Loo D. D. (2005) Kinetics of the reverse mode of the Na+/glucose cotransporter. J. Membr. Biol. 204, 23–32 16007500 10.1007/s00232-005-0743-x PMC3000923

[34] Santer R., Groth S., Kinner M., Dombrowski A., Berry G. T., Brodehl J., Leonard J. V., Moses S., Norgren, S., Skovby F., Schneppenheim R., Steinmann B., Schaub J. (2002) The mutation spectrum of the facilitative glucose transporter gene SLC2A2 (GLUT2) in patients with Fanconi-Bickel syndrome. Hum. Genet. 110, 21–29 11810292 10.1007/s00439-001-0638-6

[35] Burcelin R., del Carmen Munoz M., Guillam M. T., Thorens B. (2000) Liver hyperplasia and paradoxical regulation of glycogen metabolism and glucose-sensitive gene expression in GLUT2-null hepatocytes. Further evidence for the existence of a membrane-based glucose release pathway. J. Biol. Chem. 275, 10930–10936 10753892 10.1074/jbc.275.15.10930

[36] Guillam M. T., Burcelin R., Thorens B. (1998) Normal hepatic glucose production in the absence of GLUT2 reveals an alternative pathway for glucose release from hepatocytes. Proc. Natl. Acad. Sci. U. S. A. 95, 12317–12321 9770484 10.1073/pnas.95.21.12317 PMC22829

[37] Burcelin R., Dolci W., Thorens B. (2000) Glucose sensing by the hepatoportal sensor is GLUT2-dependent: *in vivo* analysis in GLUT2-null mice. Diabetes 49, 1643–1648 11016447 10.2337/diabetes.49.10.1643

[38] Chandra P., Brouwer K. L. (2004) The complexities of hepatic drug transport: current knowledge and emerging concepts. Pharmaceut. Res. 21, 719–735 10.1023/b:pham.0000026420.79421.8f 15180326

[39] Wong H. Y., Law P. Y., Ho Y. Y. (2007) Disease-associated Glut1 single amino acid substitute mutations S66F, R126C, and T295M constitute Glut1-deficiency states in vitro. Mol. Genet. Metab. 90, 193–198 17052934 10.1016/j.ymgme.2006.09.002

[40] Hirayama B. A., Lostao M. P., Panayotova-Heiermann M., Loo D. D., Turk E., Wright E. M. (1996) Kinetic and specificity differences between rat, human, and rabbit Na+-glucose cotransporters (SGLT-1). Am. J. Physiol. 270, G919–G926 8764197 10.1152/ajpgi.1996.270.6.G919

