# Supplementary material for: Facilitative plasma membrane transporters function during ER transit
Source: FASEB J. 2010 Aug;24(8):2849–58. doi: 10.1096/fj.09-146472 (PMC3230527; doi:10.1096/fj.09-146472)
Supplement: Supplemental Data [file fj.09-146472_index.html]

 Facilitative plasma membrane transporters function during ER transit -- Takanaga and Frommer 24 (8): 2849 Data Supplement - Supplemental Data -- The FASEB Journal **Facilitative plasma membrane transporters function during ER transit**  
*FASEB J.* Takanaga and Frommer 24: 2849

## Supplemental Data

**Files in this Data Supplement:**

- Supplemental Data - (*09-146472Supp Data.zip; compressed file 7.5 MB*)
